# Optical Coherence Tomography: Focus on the Pathology of Macula in Scleritis Patients

**DOI:** 10.3390/jcm12144825

**Published:** 2023-07-21

**Authors:** Lilla Smeller, Edit Toth-Molnar, Nicolette Sohar

**Affiliations:** Department of Ophthalmology, University of Szeged, 6720 Szeged, Hungary; toth-molnar.edit@med.u-szeged.hu (E.T.-M.); sohar.nicolette@med.u-szeged.hu (N.S.)

**Keywords:** scleritis, optical coherence tomography, macular edema, epiretinal membrane

## Abstract

Optical coherence tomography (OCT) is a non-invasive imaging technique for high-resolution, cross-sectional tissue imaging of the eye. During the past two and a half decades, OCT has become an essential tool in ophthalmology. It is a painless method for examining details of ocular structures in vivo with high resolution that has revolutionized patient care following and treating scleritis patients. Methods: Twenty-four patients diagnosed with scleritis were selected for this study. All of the patients went through basic ophthalmological examinations, such as visual acuity testing (VA), intraocular pressure measurement (IOP), slit lamp examination, ophthalmoscopic examination, and OCT. OCT examinations were taken by SD-OCT Spectralis OCT system (Heidelberg Engineering, Heidelberg, Germany). Results: Twenty-seven eyes of 24 patients (7 males and 17 females) were included in this study, who were diagnosed with scleritis. OCT examinations showed epiretinal membrane (ERM) in three patients (12%), cystoid macular edema (CME) (three cases, 12%), diffuse macular edema (DME) (one case, 4%), and serous retinal detachment (SRD) (one case, 4%). Conclusions: OCT proved to be a valuable, non-invasive method for detecting macular pathology in patients with scleritis. Despite the best treatment regimen applied, macular involvement resulting in reduced visual acuity (VA) can develop, which we could detect with OCT since macular edema (ME) is the leading cause of decreased vision due to the damaged outer blood–retina barrier (BRB) in inflammation. OCT investigation is a highly important method for early detection of ocular complications in scleritis in order to prevent blindness.

## 1. Introduction

Scleritis is a chronic and painful, vision-threatening inflammatory disease that is characterized by edema and cellular infiltration of the scleral and episcleral tissues. The most common etiology is inflammatory (noninfectious in 90% of all scleritis patients), either idiopathic or in the context of systemic disease. Scleritis is commonly associated with systemic autoimmune disorders [1], including rheumatoid arthritis, systemic lupus erythematosus, relapsing polychondritis, spondylarthropathies, granulomatosis with polyangiitis, formerly known as Wegener granulomatosis, polyarteritis nodosa, and giant cell arteritis [1,2,3].

**Figure 1:**
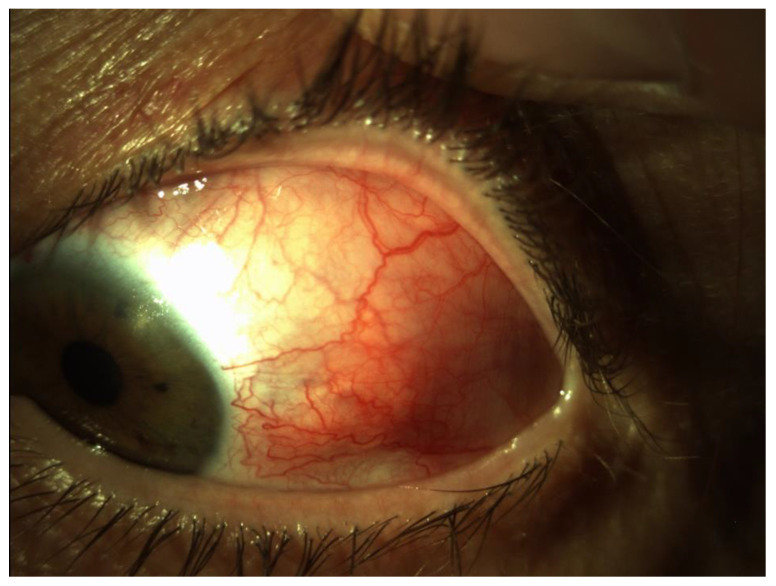
Anterior segment picture of diffuse scleritis of the temporal part of the left eye.

Scleritis may be classified as anterior and posterior ones based on the anatomical location of the inflammation. The most common clinical forms are diffuse scleritis and nodular scleritis [3] (Figure 1). Necrotizing scleritis is much less frequent and associated with systemic autoimmune disorders. Posterior scleritis is characterized by flattening of the posterior aspect of the globe, thickening of the posterior coats of the eye, and retrobulbar edema [4,5].

Scleritis is a rare disease. Although well-defined incidence rates are hard to find, the prevalence is estimated to be six cases per 10,000 people. Anterior scleritis is demonstrated in 94% of the cases, and posterior scleritis is diagnosed only in 6% of the patients [3,5,6,7]. In differential diagnosis, episcleritis is of utmost importance as it refers to the inflammation of the superficial episcleral tissue. Episcleritis is usually idiopathic, poses no serious threat to vision, and does not affect the adjacent tissues in the eye. Vessels have a reddish hue compared to the deeper-bluish hue in scleritis [3,5,6,7].

Ocular complications of scleritis, which cause vision loss and eye destruction, appear as a result of the extending scleral inflammation [3,8]. Scleritis is usually painful and can lead to vision loss due to progressive inflammation of the ocular tissues or even morbidity and mortality due to an underlying collagen vascular disease [1,2,3].

The etiology of scleritis remains unclear. Scleritis is commonly associated with systemic autoimmune disorders and systemic vasculitis. Immunohistochemistry studies reveal that there is a localized scleral vasculitis, most likely secondary to the deposition of circulating immune complexes, in patients with necrotizing scleritis [9,10].

Scleritis may often pose a diagnostic challenge since the clinical features are subtle and diagnostic modalities are limited [1,2]. The diagnosis of scleritis is usually based on clinical assessment and ultrasonography. B-scan ultrasound is the most useful confirmatory analysis for posterior scleritis diagnosis [11]. It can show the diffuse thickness of the choroid because of the increased amount of fluid in subtenon space and around the optic nerve, the so-called T sign [3].

The variability in clinical presentations and also in ultrasonography findings, as well as unfamiliarity with the diagnosis, account for the fact that scleritis is one of the most underdiagnosed conditions in ophthalmology [3,7].

Generally, scleritis requires systemic therapy. Nonsteroidal anti-inflammatory drugs (NSAIDs), corticosteroids, or immunomodulatory drugs can be indicated. Topical therapy is routinely insufficient. The treatment must be individualized according to the severity of scleritis, response to treatment, adverse effects, and presence of associated diseases [3,12,13,14]. Oral corticosteroids (1 mg/weight kg) supplemented with periorbital and subconjunctival steroid injections were the first therapeutic regimen introduced [12,13]. In case of therapeutic failure of corticosteroids, immunosuppressive drugs were added. Methotrexate (MTX) [15] was the first choice, but azathioprine, cyclophosphamide [16], and cyclosporine [17] were also helpful. In other cases, other immunomodulatory drugs were effective, such as biologics, which were ordered by rheumatologists [13]. Scleritis may pose a diagnostic challenge since the clinical features are subtle and diagnostic modalities are limited.

More recently, tumor necrosis factor (TNF) alpha inhibitors such as infliximab have shown promise in the treatment of non-infectious scleritis refractory to other treatments. This consists of regularly repeated infusions since the treatment effect is short-lived. However, TNF-alpha inhibitors may also result in a drug-induced lupus-like syndrome (that can also generate ophthalmic disorders) as well as an increased risk of lymphoproliferative disease. All patients on immunomodulatory therapy must be strictly monitored by rheumatologists to avoid systemic complications with the medication [13].

Optical coherence tomography (OCT) has become the most important non-invasive diagnostic technique to evaluate ophthalmic pathologies, also involving the macula with cross-sectional tissue imaging [18,19]. Since the introduction of OCT in 1991, it has become an essential tool in ophthalmology [20].

It is a non-contact, painless method for detailing ocular structures in vivo with high resolution. OCT uses light in the near-infrared spectral range (in the 800–840 nm wavelength range) and penetrates at a depth of several hundred microns in the tissue. It provides real-time, non-invasive imaging of the retina. OCT can be used to follow and reproduce quantitative and qualitative retinal thickness [21,22]. OCT images correlate well with retinal histology [23].

With OCT datasets, we can give exact information about the dynamics of disease progression and response to treatment based on analyzing the retinal anatomy. OCT is also suitable for detecting and monitoring uveitic macular edema and the changes in the fluid distribution in eyes with macular edema, as well as detecting the morphology of the vitreoretinal interface [21,22,23,24].

OCT is based on the principle of Michelson interferometry, where a low-coherence light beam is directed at the target tissue, and the scattered back-reflected light is combined with a second beam (reference beam). The resulting interference patterns are used to reconstruct an axial A-scan, which represents the beam’s path. From all of the A-scans (time amplitude scan), a two-dimensional cross-sectional image of the target tissue can be reconstructed, called B-scan (brightness amplitude scan). These B-scans are repeated at multiple adjacent positions using a raster scan pattern, then a three-dimensional volume of structural and flow information can be structured [21,22]. The scanning beam allows for the acquisition of cross-sectional images of the tissue structure. Light source and detector characteristics determine the axial resolution and imaging range of an OCT, not the focusing optics. [21,22].

Normal retinal tissue has different reflectivity patterns on OCT. The nerve fibers and the retinal pigment epithelium display high, the plexiform and the nuclear layers display medium, and the photoreceptors display low reflectivity [25].

The OCT measures retinal thickness automatically. The distance between the vitreoretinal interface and the anterior surface of the retinal pigment epithelium is generally 200–275 μm, and the foveal depression has a range from 170 to 190 μm. The axial resolution is 3.9 µm/pixel, and the lateral resolution is 5.7 µm/pixel. Using several algorithms, cube scans also allow measurement of the volume of the macula [26].

The image acquisition time is limited by the patient’s ability to avoid eye movements and the availability of tracking software that adjusts for eye movements. We use spectral domain OCT (SD-OCT); its scanning speed can exceed 100,000 A-scans per second. SD-OCT systems operate at scanning rates of approximately 27,000–70,000 A-scans per second. As the A-scan density increases, resolution becomes higher, and SD-OCT produces better-quality B-scans. Higher scanning speed reduces the effect of artifacts made by eye motion and produces images that provide a true picture of the retina [27]. The large, dense raster scans make it possible to obtain detailed surfaces of individual retina layers over large areas, resulting in segmentation maps [28,29].

With OCT datasets, we can give exact information about the dynamics of disease progression and response to treatment based on analyzing the retinal anatomy [21,22].

OCT imaging also has limitations; as OCT utilizes light beams, media opacities can interfere with optimal imaging in spite of ultrasound’s sound waves. Patient cooperation is necessary as eye and patient movement can diminish the image’s quality.

In posterior scleritis, different manifestations of choroidal involvement are known on OCT: increased choroidal thickness, choroidal vasculitis, presentation as a choroidal or subretinal mass in nodular posterior scleritis, and choroidal folds, choroidal effusion and exudative retinal detachment [6,11].

Macular edema (ME) is defined as a thickening of the macular region caused by the breakdown of the outer and/or inner blood–retina barrier leading to increased permeability of the retinal pigment epithelium and the retinal vasculature. The leakage from perifoveal capillaries results in the accumulation of intracellular and extracellular fluid [18]. ME can persist or recur despite improvement or resolution of the ocular inflammation [18,24,30]. Visual acuity could be decreased because of ME due to impaired cell function relationships in the retina [31].

**Figure 2:**
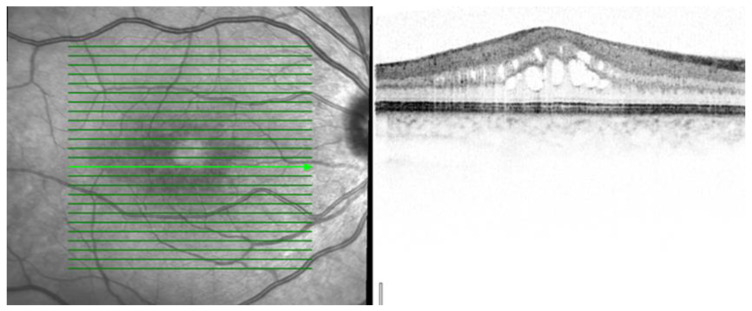
OCT of the right eye of a 28-year-old woman showing CME with loss of the foveal depression and intra-retinal cysts in the outer and inner nuclear layer of the retina. OCT scale: 512 × 496, high-speed mode, 20°.

**Figure 3:**
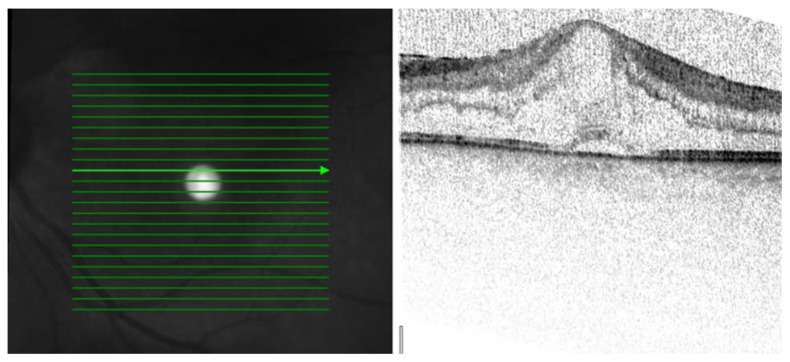
On this OCT image: DME is characterized by the disturbance of the layered retinal structure or low reflective areas looking similar to a sponge. The complication of corticosteroid therapy is cataract formation which can explain the quality of the image. OCT scale: 512 × 496, high-speed mode, 20°.

**Figure 4:**
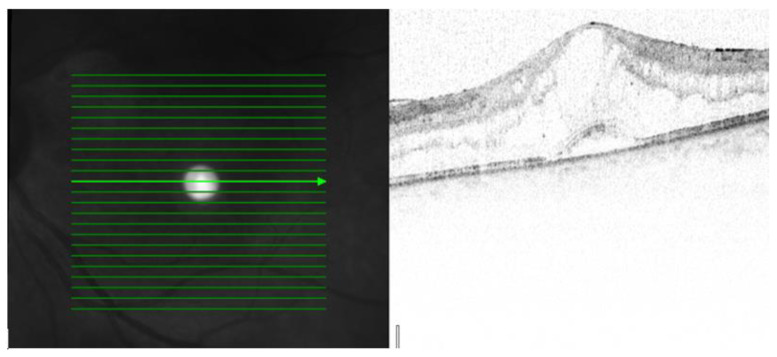
OCT image from left eye: In SRD, fluid accumulates in the subretinal space between the sensory retina and the retinal pigment epithelium. OCT scale: 512 × 496, high-speed mode, 20°.

Three patterns of macular edema can be revealed: Cystoid macular edema (CME) is the formation of fluid-filled cyst-like spaces between the outer plexiform and inner nuclear layer of the retina. Diffuse macular edema (DME) is characterized by the disturbance of the layered retinal structure or low reflective areas looking similar to a sponge. In serous retinal detachment (SRD), fluid accumulates in the subretinal space between the sensory retina and the retinal pigment epithelium [32] (Figure 2, Figure 3 and Figure 4).

Symptoms of macular edema include metamorphopsia, micropsia, blurred vision, a central scotoma, and reduction in contrast or color sensitivity. The clinical diagnosis of macular edema can be challenging in mild cases or when visualization of the fundus is impaired by poor pupillary dilation, corneal disorders, cataract, vitreous hemorrhage, and other ocular media opacities [30].

Causes of fluid accumulation include inflammatory, infectious, and neoplastic diseases of the choroid or retina. Retinal dystrophies and other retinal vascular abnormalities, including retinal arterial macroaneurysms and retinal telangiectasia, can also cause different types of edema in the macula.

Cystoid macular edema (CME) represents a common pathologic change in the retina and occurs in a variety of pathological conditions, such as intraocular inflammation. In pars planitis, there is an accumulation of T-cell inflammatory mediators associated with CME. ME may appear in central or branch retinal vein occlusion, diabetic retinopathy, and most commonly following cataract extraction (Irvin–Gass syndrome). E2-prostaglandins can also cause disruption of the tight junctions of the retinal capillaries causing CME. Niacin or nicotine acid intoxication can also be a rare cause of CME [18,21].

Diffuse retinal thickening is the main pattern in diabetic macular edema.

Serous detachments, with the elevation of the retina, occur in a variety of disorders, including central serous chorioretinopathy (CSC), age-related macular degeneration (AMD), systemic lupus erythematosus (SLE), and choroidal ischemic disorders, such as accelerated hypertension, pre-eclampsia, eclampsia, systemic corticosteroid usage, or in some choroidal tumors and inflammatory disorders, for example, in Vogt–Koyanagi–Harada’s disease [18].

When ME is found on OCT images, the therapy has to be extended or changed to save vision. In persistent ME and decreased best corrected visual acuity (BCVA), local therapy is needed, such as non-steroid drops. If this treatment is ineffective, corticosteroid injection is needed, either intravitreally or sub-tenonly, or an intravitreal steroid implant should be applied in order to treat this complication.

**Figure 5:**
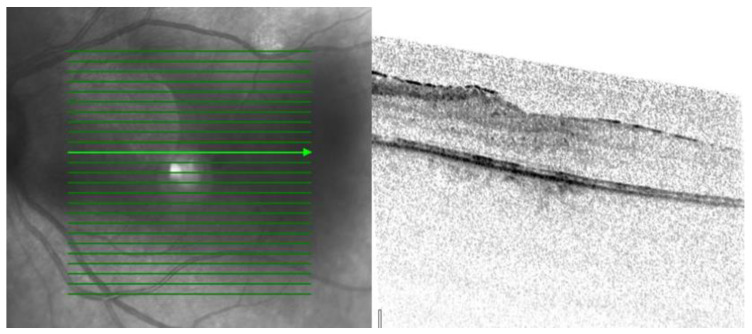
OCT image of the epiretinal membrane (ERM): a reflective layer on the top of the internal limiting membrane (ILM) of a 28-year-old patient. The ERM is attached to the retinal surface of the left eye. OCT scale: 512 × 496, high-speed mode, 20°.

On OCT images, ERM appears as a hyperreflective line adhering to the retina. In recent studies, secondary ERMs were associated with worse visual acuity in comparison to idiopathic ERM. This complication is commonly seen in recurrent uveitis or other ocular inflammation [33] (Figure 5). Its therapy is epiretinal membrane peeling with a pars plana vitrectomy procedure [34].

Biomarker’s definition by the United States National Institutes of Health (NIH) is “a characteristic that is measured as an indicator of normal biological processes, pathogenic processes or responses to an exposure or intervention, including therapeutic interventions” [35].

The aims of this study were to investigate scleritic patients with basic ophthalmological examination methods and with OCT in order to find any macular complications and also to determine whether the macular complications could affect the prognosis and the treatment and become biomarkers since previously, macula was not examined in scleritis patients.

## 2. Materials and Methods

Twenty-seven eyes of 24 patients (7 males and 17 females) were included in this study, who were diagnosed with non-infectious scleritis. The mean age was 57.75 years (range: from 30 to 77 years).

Scleritis was diagnosed by the presence of the following parameters: (1) acute or sub-acute symptom onset; (2) eye pain with or without decreased visual acuity; (3) posterior sclerochoroidal wall thickening. Scleritis was classified as diffuse, nodular, or necrotizing. The location of inflammation was also recorded.

First, all of the patients went through basic ophthalmological examinations such as visual acuity (VA) testing. Visual acuity was tested by the Early Treatment of Diabetic Retinopathy Study (ETDRS) chart, intraocular pressure measurement (Goldman tonometry), slit lamp examination, indirect ophthalmoscopic examination, and OCT.

OCT examinations were taken by SD-OCT Spectralis OCT system (Heidelberg Engineering, Heidelberg, Germany, Software version: Heidelberg Eye Explorer 1.9.13.0).

OCT scan parameters were as follows: infrared scan; pattern size: 20° × 20°; 25 sections; 240 µm between B-scans; 512 A-scans.

OCT examination was performed in all of the cases at the time of their presentation at our uveitis outpatient clinic. For standardization, all examinations were performed by the same technician. The thickness of the retina was measured between the inner limiting membrane and Bruch’s membrane in the central macular region. In addition to these investigations, all participants were subjected to laboratory tests and rheumatological examinations to find out if there were any associated systemic diseases.

This research was conducted ethically in accordance with the World Medical Association Declaration of Helsinki. In this manuscript, we, the authors, state that subjects have given their written informed consent to publish their case (including publication of images). Information revealing the subject’s identity is avoided. All patients can be identified by numbers or aliases and not by their real names.

Human subject research has been performed with the approval of the Regional and Institutional Review Board of Human Investigations at the University of Szeged and with appropriate participants’ informed consent in compliance with the Helsinki Declaration.

This study’s protocol was reviewed and approved by the Regional and Institutional Review Board of Human Investigations at the University of Szeged, approval number 5053. Date of approval 20 January 2020.

We state that written and signed informed consent was obtained from all participants for publication of the details of their medical cases and any accompanying images.

## 3. Results

The demographic and clinical data of the 24 patients are shown in Table 1. Three patients had bilateral disease. Thirteen patients (54%) had associated systemic disease: rheumatoid arthritis (*n* = 5); granulomatosis with polyangiitis, formerly known as Wegener granulomatosis (*n* = 1); ulcerative colitis (*n* = 1); collagenosis (*n* = 1); dermatopolymiositis (*n* = 1); pemphigoid (*n* = 1); non-differentiated collagenosis (NDC) syndrome (*n* = 1); ankylosing spondylitis (*n* = 1); and Cogan syndrome (*n* = 1). There were twenty-four anterior and three posterior scleritis.

Among the twenty-four eyes diagnosed with anterior scleritis, there were 16 with diffuse scleritis and 8 with nodular anterior scleritis. One patient had peripheral ulcerative keratitis; one had retinal detachment, and one had hydro-keratopathy.

Table 2 presents the data dealing with the macula. After investigating the patients, five of them (18.5%) had macular disorders. Their visual acuities were below 37 + 30 letters on the early treatment of diabetic retinopathy study (ETDRS) chart. OCT demonstrated three patterns of macular edema in the examined patients CME three cases (12%), DME one case (4%), and SRD one case (4%).

The overall mean VA of all the scleritic patients was 28 + 30 letters with correction, and the mean retinal thickness at the central fovea was 291.7 μm in our patients.

The mean VA was 22 + 30 letters in patients with CME, 19 + 30 letters in patients with DME, and 33 + 30 letters in our patients with SRD. The mean CRT was 558 μm in patients with CME, 328 μm in patients with DME, and 288 μm in our patients with SD. The central retina thickness (CRT) was the thickest in cases of CME and thinnest in cases of SRD.

The macular thickness, as seen on OCT, is objective and correlates with BCVA.

The patients with CME were treated with triamcinolone (TA) injection sub-tenonly when topical non-steroid eye drops were ineffective.

OCT examinations showed ERM in three patients (12%).

None of our patients with ERM have gone through vitrectomy surgery so far due to the close OCT follow-up.

## 4. Discussion

Although scleritis is a rare disease characterized by inflammation of the sclera and adjacent ocular structures, its complications are vision-threatening. Studies have led to significant progress in understanding the epidemiology, immunopathogenesis, severity assessment, treatment, and prognosis of this potentially sight-threatening disease [3,12].

OCT has the advantage of being a fast and noninvasive imaging technique that provides a quantitative assessment of the macular thickness to monitor the clinical course and helps make therapeutic decisions. We can follow the activity of the disease and the response to therapy. It is suitable for detecting and monitoring inflammatory macular edema, the changes in fluid distribution in these cases, and the morphology of the vitreoretinal interface [18,36].

Using OCT, we could find the adequate diagnosis leading to the best, complex treatment and follow-up of the disease. OCT can give more information about the depth of the inflammation and the prediction of visual outcomes than previous examinations such as ultrasound, for example. The thickness of the retina and macula measured by OCT is a potential indicator of retinal inflammation. Four studies used OCT to measure retinal/macular thickness and compared it with the presence of retinal vasculitis. The results suggest that increased retinal/macular thickness correlates with retinal vasculitis. [37]. There is no report in the literature about using OCT to detect any macular entity in cases of scleritic patients.

The most common and vision-decreasing complication of inflammation is ME, which can persist or recur despite improvement or resolution of the ocular inflammation. ME is an important cause of reduced visual acuity in many retinal diseases, for example, diabetic retinopathy, retinal vein occlusion, age-related macular degeneration, uveitis, and following intraocular surgery. ME always appears as retinal thickening with intraretinal cavities of reduced reflectivity on OCT [30].

Kempen et al. described the use of OCT in patients with uveitis and tried to detect early ME [24]. Markomichelakis et al. defined uveitic ME and noted three different patterns of fluid accumulation that were the same as in diabetic ME: diffuse macular edema (DME), cystoid macular edema (CME), and serous retinal detachment (SRD) [32].

Iannetti et al. also reported OCT findings in patients with ME from uveitis—58% had CME, and 42% had DME. SRD was noted in 28% of all cases [38]. Comparing the OCT data of our series, we diagnosed three patients with CME (12%), one patient (4%) with DME, and one patient (4%) with SRD among all scleritic patients.

CME and DME lead to reduced VA, which can affect patients’ quality of life. The vision was 22 + 30 in the case of CME and 19 + 31 in DME. The central retina thickness was the thickest in cases of CME and the least thick in cases of SRD.

The visual impact of ERM in eyes with inflammation is not clear. The presence of ERM in the macular area suggests the hypothesis of tractional mechanism as an origin or cofactor of appearing macular edema during inflammatory diseases. ERM can be independent of the type of macular edema and the type of inflammation [34,36,39].

It is known that in most eyes with inflammatory ERM, visual acuity remains stable if intraocular inflammation and co-morbidities are treated appropriately [33,40]. ERM occurs in approximately 6% of patients over the age of 60 [33,40]. Although none of the patients was older than 60 years in our study, OCT examinations showed ERM in three patients (12%), and their vision remained stable at 45 + 30.

ERMs can be classified as idiopathic or secondary in an already-existing ocular pathology. Most idiopathic ERMs are thought to result from fibroglial proliferation on the inner surface of the retina secondary to a break in ILM during posterior vitreous detachment. Glial cells, retinal pigment epithelium (RPE) cells, and myofibroblasts are shown to be mostly involved in ERM formation [33].

On OCT, ERMs are seen as a highly reflective layer on the inner retinal surface. The membrane is adherent to the retina, but sometimes, it can be separated from the inner aspect of the retina, which enhances its visibility by OCT. Secondary effects of the membrane can be the loss of the normal foveal contour, increased retinal thickness, and the presence of cystoid changes [40].

The effectiveness of subconjunctival steroid therapy and the introduction of highly effective systemic immunosuppressive drugs and biologicals have had a significant impact on controlling this potentially blinding and painful inflammatory eye disease [41]. The macular complications influenced the visual prognosis and the treatment as well.

The main cause leading to macular edema is the breakdown of either the inner or outer, or both blood–retina barriers (BRB) and is a consequence of chronic inflammation. Extracellular fluid is accumulated either in the intraretinal or the subretinal space. The macular edema can be found in the outer nuclear layer or extend more superficially or deeply before affecting all retinal layers. This results from the sum-up of cytotoxic and vasogenic effects due to immunological aggression [30].

The outer (BRB) is important for maintaining the adhesion between the retinal pigment epithelium (RPE) and photoreceptors. Inflammatorical conditions that involve the sclera could damage the outer BRB, and despite the healthy retinal capillary endothelium, macular edema might occur. Other causes may also increase the macular thickness, such as inflammatorical ERM formation with associated vitreomacular traction [30].

TNF can also have a significant part in the pathogenesis of ME. Infliximab is currently licensed to be used as a third-line agent in scleritis therapy following the development of tolerance or failure to respond to first-line corticosteroid and to second-line corticosteroid-sparing agents. Until now, it is not proven that the earlier introduction of anti-TNF agents would give additional benefits to the management of ME and the preservation of visual function [10]. It is proven that inhibiting the level of TNF-α decreases the incidence of ME [10,14].

We can state that OCT findings help ophthalmologists determine visual outcomes. ME and ERM can cause worsening of vision, but visual improvement can be achieved by systemic and additional ophthalmologic therapy. CME, SRD, DME, and ERM negatively affect VA. Especially in chronic scleritis cases, ME and ERM could work as biomarkers since biomarkers provide an objective, measurable method of evaluating the disease process. Some biomarkers can help researchers to identify the risk factors of the disease [35]. The photoreceptor layer is concerned with the fact that, because of the long-standing (chronic or persistent) or frequently recurrent pathologies, visual acuity will not be better in spite of the best therapy.

The limitation of our study is the low number of patients, as the prevalence of scleritis is six cases per 10,000 people.

## 5. Conclusions

OCT has revolutionized patient care because it allows early, accurate diagnosis and better follow-up of patients. To the best of our knowledge, this is the first report that investigates macula in anterior scleritic patients. We detected OCT examinations of all of our patients since we thought that OCT would be a suitable, non-invasive method to detect macular pathology, and our results proved that it was. We found that the reduced VA in cases of scleritis patients could be the consequence of macular involvement.

In conclusion, besides treating scleritis patients, the examination of their macula by OCT is also very important in order to detect any macular complications caused by inflammation since CME is the leading cause of decreased vision. ERM is also associated with poor vision. Macular pathologies seen on OCT can modify the management of scleritis.

OCT plays an important role in measuring inflammatorical activity, determining the severity of inflammation, choosing the best treatment, the response to treatment, and avoiding legal blindness. Biomarkers on OCT in scleritic patients, such as CME, DME, SRD, and ERM, are also very useful in order to find the right diagnosis and treatment in time.

## Figures and Tables

**Table 1:** Demographic and clinical data of our scleritis patients.

Age (years)	30–77	
Mean age (years)	57.75	
Gender	7 male: 17 female	
Laterity	21 unilateral: 3 bilateral	
Background of scleritis		
	rheumatoid arthritis	5
	Wegener granulomatosis	1
	ulcerative colitis	1
	collagenosis	1
	dermatopolyomiositis	1
	pemphigoid	1
	non-differentiated collagenosis	1
	ankylosing spondilitis	1
	Cogan-syndrome	1
	unknown etiology	11

**Table 2:** OCT findings in our patients.

OCT Findings	Number of Patients (% of Total no = 24)	CRT (μm)	VA (ETDRS)
cystoid macular edema	3	558	22 + 30
	(12%)		
diffuse macular edema	1	328	19 + 30
	(4%)		
serous retinal detachment	1	288	33 + 30
	(4%)		
epiretinal membrane	3	402	45 + 30
	(12%)		

## Data Availability

The authors confirm that the data supporting the findings of this study are available within the article.

## Abbreviations

AMD	age-related macular degeneration
BCVA	best corrected visual acuity
BRB	blood–retina barrier
CME	cystoid macular edema
CRT	central retina thickness
DME	diffuse macular edema
ERM	epiretinal membrane
ETDRS	early treatment of diabetic retinopathy study
ME	macular edema
MTX	methotrexate
NIH	National Institution of Health
OCT	optical coherence tomography
RPE	retinal pigment epithelium
SLE	Systemic Lupus Erythematosus
SRD	serosus retinal detachment
TA	triamcinolone-acetate
VA	visual acuity
